# Physiological Analysis of Source–Sink Relationship in Rice Genotypes with Contrasting Grain Yields

**DOI:** 10.3390/plants13010062

**Published:** 2023-12-23

**Authors:** Chandrapal Vishwakarma, Gopinathan Kumar Krishna, Riti Thapar Kapoor, Komal Mathur, Monika Dalal, Nagendra Kumar Singh, Trilochan Mohapatra, Viswanathan Chinnusamy

**Affiliations:** 1 Division of Plant Physiology, Indian Council of Agricultural Research-Indian Agricultural Research Institute (IARI), New Delhi 110012, India; chandrapalv29@gmail.com; 2 Amity Institute of Biotechnology, Amity University Uttar Pradesh, Noida 201313, Uttar Pradesh, India; rkapoor@amity.edu (R.T.K.); komalsuhani1972@gmail.com (K.M.); 3 Department of Plant Physiology, College of Agriculture, Vellanikkara, Kerala Agricultural University, Thrissur 680656, Kerala, India; krishna.kg@kau.in; 4 Indian Council of Agricultural Research-National Institute for Plant Biotechnology, New Delhi 110012, India; monika.dalal@icar.gov.in (M.D.); nk.singh@icar.gov.in (N.K.S.); 5 Protection of Plant Varieties and Farmers’ Rights Authority, New Delhi 110012, India; tmnrcpb@gmail.com

**Keywords:** rice (*Oryza sativa L.*), photosynthesis, source–sink, grain, leaf, starch and sugar, gene expression, allele, genes and traits

## Abstract

Rice is a major staple food, and, hence, doubling its productivity is critical to sustain future food security. Improving photosynthesis, source–sink relationships and grain-filling mechanisms are promising traits for improvement in grain yield. To understand the source–sink relationship and grain yield, a set of contrasting rice genotypes differing in yield and biomass were studied for physiological, biochemical and gene-expression differences. The physiological and yield component traits of selected rice genotypes were analyzed in 2016 and 2017 under field conditions. This led to the categorization of genotypes as high yielding (HY) and high biomass, viz., Dular, Gontra Bidhan 3, Way Rarem, Patchai Perumal, Sahbhagi Dhan, Indira Barani Dhan-1, MTU1010, and Maudamani; while, low yielding (LY) and low biomass, viz. Anjali, Ghanteswari, Parijat, Khao Daw Tai, RKVY-104, Ghati Kamma Nangarhar, BAM4510 and BAM5850. The HY genotypes in general had relatively better values of yield component traits, higher photosynthetic rate (Pn) and chlorophyll (Chl) content. The study revealed that leaf area per plant and whole plant photosynthesis are the key traits contributing to high biomass production. We selected two good-performing (Sahbhagi Dhan and Maudamani) and two poor-performing (Ghanteswari and Parijat) rice genotypes for a detailed expression analysis of selected genes involved in photosynthesis, sucrose synthesis, transport, and starch synthesis in the leaf and starch metabolism in grain. Some of the HY genotypes had a relatively high level of expression of key photosynthesis genes, such as *RbcS*, *RCA*, *FBPase*, and *ZEP* over LY genotypes. This study suggests that traits, such as leaf area, photosynthesis and grain number, contribute to high grain yield in rice. These good-performing genotypes can be used as a donor in a breeding program aimed at high yields in rice.

## 1. Introduction

Rice (*Oryza sativa L*.) is the staple food crop for over half of the world’s population. Rice is considered a model-crop plant that can be easily studied due to its diploid genetic nature (2 *n* = 24), small genome size (389 Mb), short generation time, and high genetic polymorphism [1,2]. Further, rice is cultivated across diverse ecological, geographical, and agronomic conditions [3]. Dwindling freshwater resources and climate change are the major challenges to enhancing global crop productivity by 50% to ensure food security by 2050 [4]. To achieve this goal, improving photosynthesis, source–sink relationships, and grain-filling mechanisms are promising strategies [5].

Photosynthesis is a major plant physiological process on Earth that synthesizes carbohydrates using CO_2_, water, and sunlight. Photosynthesis at the leaf level and whole canopy level constitutes the primary “source”, and photoassimilates stored in different tissues constitute a secondary source. From these sources, metabolites are transported to “sink” tissues, that is grains, and, thus, determine the harvestable yield crop plants. Optimization of the source–sink balance is important to break the yield ceiling. The major source that contributes to rice grain development is current photosynthesis followed by nonstructural carbohydrates stored in the stem, leaf sheath, and leaves. Hence, high biomass accumulation at anthesis and maintenance of leaf area are important to provide carbohydrates for the developing sink, i.e., grains [6,7]. In addition to having an optimum source size, the presence of a strong sink (number of grains per panicle and grain weight) is essential to achieve a high yield in rice. It has been demonstrated earlier that an increase in sink strength (number of spikelets per panicle and grain size) leads to an increase in grain yield in rice [8,9,10]. Poor grain filling was identified as the cause for the failure to achieve greater yield in hybrids of japonica/indica rice, which produce a huge sink [11]. For enhanced source strength, and thus yield in rice, high biomass and high N content at flowering and better canopy structure for postanthesis radiation-use efficiency (RUE) are suggested [7].

As current photosynthesis is the key component of source strength, the optimization of unit leaf photosynthesis and canopy photosynthesis is critical for improving source strength in rice [7]. In rice, a transcription factor HYR (higher yield rice) was identified as a master regulator which increased photosynthesis in different environmental conditions and improved grain yield [12]. The photosynthesis of the crops can be increased through optimization of the leaf area, increase in leaf-area duration, daily duration of leaf photosynthesis, and rate of photosynthetic per unit leaf area per unit time [7,13].

To break the yield barriers, China initiated a “super rice” megaproject in 1996, which resulted in the release of more than 131 “super rice” varieties with grain yields of more than 12 t ha^−1^ [14,15]. The increase in yield was attributed to an increase in sink size, higher source capacity, and higher dry-matter production [16]. Improvement in biomass is a key component for increasing source capacity. Biomass is a quantitative trait with many small additive effects and shows high variability among individuals. However, a study on QTL-based selection for biomass showed that it is effective in enhancing biomass yield in rice [16]. Rice plants overexpressing ribulose 1,5-bisphosphate carboxylase–oxygenase (Rubisco), a key enzyme for photosynthesis, also showed higher biomass and grain yield, thereby suggesting that improving photosynthesis is a promising approach to improving plant biomass [7,17,18].

In the present study, we analyzed the physiological, biochemical, and molecular responses of rice genotypes with contrasting biomass and grain yield. Based on the yield performance under field conditions during the rainy (*kharif*) season of 2016 and 2017, genotypes with contrasting grain yield per plant were selected. This study examined the relationship between photosynthesis, source size, assimilate partitioning and sink size, and also analysed the expression of genes related to the source–sink activity in rice. The best genotypes identified in this study can be used as donors for improving the yield potential of rice.

## 2. Results

### 2.1. Yield and Yield Components

The phenology, yield component, and physiological traits of contrasting rice genotypes recorded in two seasons, i.e., kharif 2016 and 2017, are presented in Supplementary Tables S1–S3. Based on the grain yield per plant, these genotypes were classified as low-yielding (LY) and high-yielding (HY) genotypes. The mean grain yield per plant in the LY group was 12.6 (±1.8) and 14.2 (±1.3) g-plant^−1^ in 2016 and 2017, respectively, while, in the HY group, it was 25.6 (±2.0) and 25.5 (±2.2) g-plant^−1^ in 2016 and 2017, respectively (Table S1). Thus, the HY group produced a 1.9-fold higher grain yield than the LY group. 

Grain yield is the fraction of total biomass that is partitioned into the grains. Hence, biomass and harvest index (HI) were analyzed. Mean biomass per plant in the LY group was 30.2 (±3.4) and 30.0 (±2.3) g-plant^−1^ in 2016 and 2017, respectively, while, in the HY group, it was 52.7 (±6.6) and 49.0 (±4.0) g-plant^−1^ in 2016 and 2017, respectively (Table S1). The mean HI (%) in the LY group was 42.2 (±5.1) and 48.1 (±5.0) g-plant^−1^ in 2016 and 2017, respectively, while in the HY group it was 49.2 (±2.5) and 51.9 (±2.6) g-plant^−1^ in 2016 and 2017, respectively (Table S1). 

Days to anthesis (DTA) of the LY genotypes ranged from 66.3 to 93.7 days, and it ranged from 79.7 to 113.3 days for the HY genotypes across years (Table S2). Days to maturity (DTM) of the LY genotypes ranged from 90.0 to 117.0 days, and it ranged from 103.3 to 136.0 days for HY genotypes across years (Table S2). 

We further analyzed the yield component, viz. panicle number per plant, grain number per panicle, grain number per plant, and 1000 grain weight. The mean panicle number (PN) -plant^−1^ was about eight and was similar between the LY and HY genotypes (Table S3). However, the grain number (GN) panicle ^−1^ showed a significant difference between the LY and HY groups of genotypes. The GN panicle^−1^ ranged from 36.0 to 102.8 and 79.3 to 201.2, respectively, among the LY and HY genotypes across two years. In the LY genotypes, the mean GN panicle ^−1^ was 76.02 (±9.77) and 75.0 (±10.4), respectively, in 2016 and 2017, while, in the HY group, it was 136.4 (±10.3) and 139.8 (±13.3), respectively, in 2016 and 2017 (Table S3). Thus, the HY groups produced a 1.9-fold higher GN panicle^−1^ compared with that of the LY group.

### 2.2. Source Traits

Biomass accumulation depends on photosynthesis, leaf area, and leaf-area duration. Here, we analyzed the photosynthesis of flag leaf, chlorophyll content, flag-leaf area, and whole-plant leaf area to understand the differences in biomass accumulation between the LY and HY groups. 

**Figure 1:**
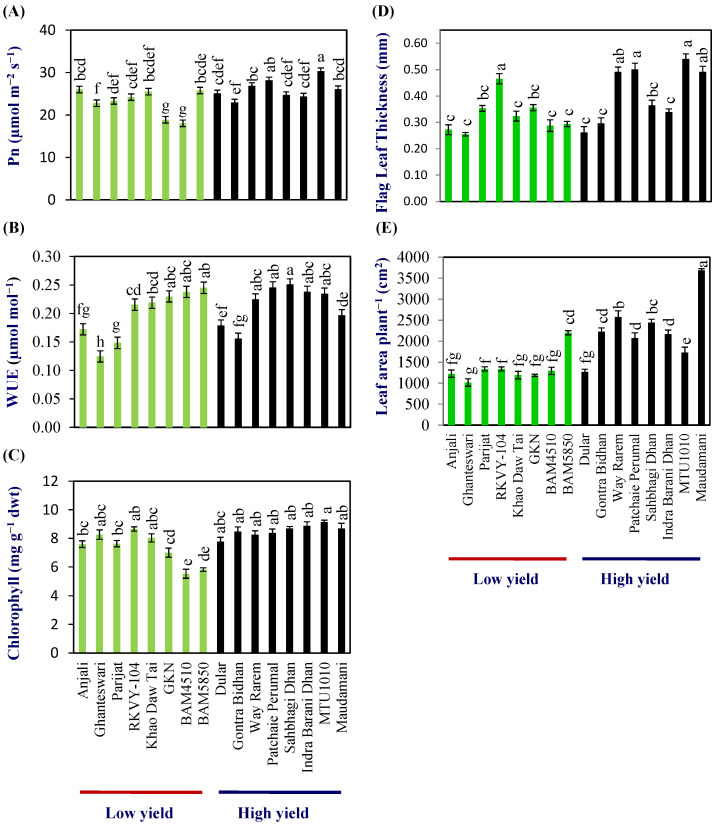
Physiological analysis in low- and high-yielding rice genotypes. (**A**) Photosynthetic rate (Pn), (**B**) water-use efficiency (WUE), (**C**) chlorophyll content (**D**) flag-leaf thickness, and (**E**) leaf area per plant. Different letters above the bars indicate statistically significant difference at *p* < 0.05.

Flag-leaf photosynthesis (Pn) at anthesis varied from 18.0–26.0 μmol m^−2^ s^−1^ with a mean of 23.2 (±0.5) μmol m^−2^ s^−1^ in the LY group and 23.0–30.3 μmol m^−2^ s^−1^ with a mean of 26.0 (±0.32) μmol m^−2^ s^−1^ in the HY group (Figure 1A; Table S4). The WUE (µmol CO_2_ mmol^−1^ H_2_O) varied from 0.13–0.25 and 0.16–0.25 in the LY and HY groups, respectively. The mean WUE (µmol CO_2_ mmol H_2_O^−1^) was 0.20 (±0.01) and 0.22 (±0.00) in the LY and HY groups, respectively, and, thus, the WUE was about 10% higher in the HY group compared with LY group at flag-leaf level (Figure 1B; Table S4).

The chlorophyll content of the flag leaf varied from 5.5 to 8.6 with a mean of 7.3 (±0.25) mg g^−1^ dry wt in the LY group, while, in the HY group, it varied from 7.7 to 9.1 with a mean of 8.5 (±0.25) mg g^−1^ dry wt in the HY group (Figure 1C; Table S4).

Similarly, significant differences were also found in the flag-leaf area. Flag-leaf area varied from 45.3 to 70.7 cm^2^ with a mean of 54.1 (±4.6) cm^2^ per flag leaf in the LY group, and 56.1–122.0 cm^2^ with a mean of 77.3 (±7.0) cm^2^ per flag leaf in HY group (Table S4). Flag-leaf thickness varied from 0.253 to 0.535 mm with a mean of 0.34 (±0.02) mm in the LY group, while, in the HY group, it varied from 0.306–0.538 mm, with a mean of 0.411 (±0.03) mm in the HY group (Figure 1D; Table S4).

The mean leaf area per plant at anthesis was 1349.23 (±65.86) and 2271.9 (±100.15) cm^2^ plant^−1^, respectively, for LY and HY groups. Thus, the total leaf area showed a clear distinction between the LY and HY groups (Figure 1E; Table S4). 

Assuming the photosynthesis rate measured on the flag leaf using IRGA is the same in the entire flag-leaf area, the calculated photosynthesis rate per flag leaf per minute [photosynthesis rate µmol per m^2^ per second × flag leaf area in m^2^ × 60 s)] varied from 6.4 to 9.1 μmol flag leaf^−1^ min^−1^ with a mean of 7.6 (±1.2) μmol. flag leaf^−1^ min^−1^ in the LY group and 7.7–20.6 μmol. flag leaf^−1^ min^−1^ with a mean of 12.58 (±4.3) μmol. flag leaf^−1^ min^−1^ in the HY group. Thus, the photosynthesis rate per flag leaf was higher in the HY group compared with the LY group. 

### 2.3. Leaf Sugar and Starch Content

**Figure 2:**
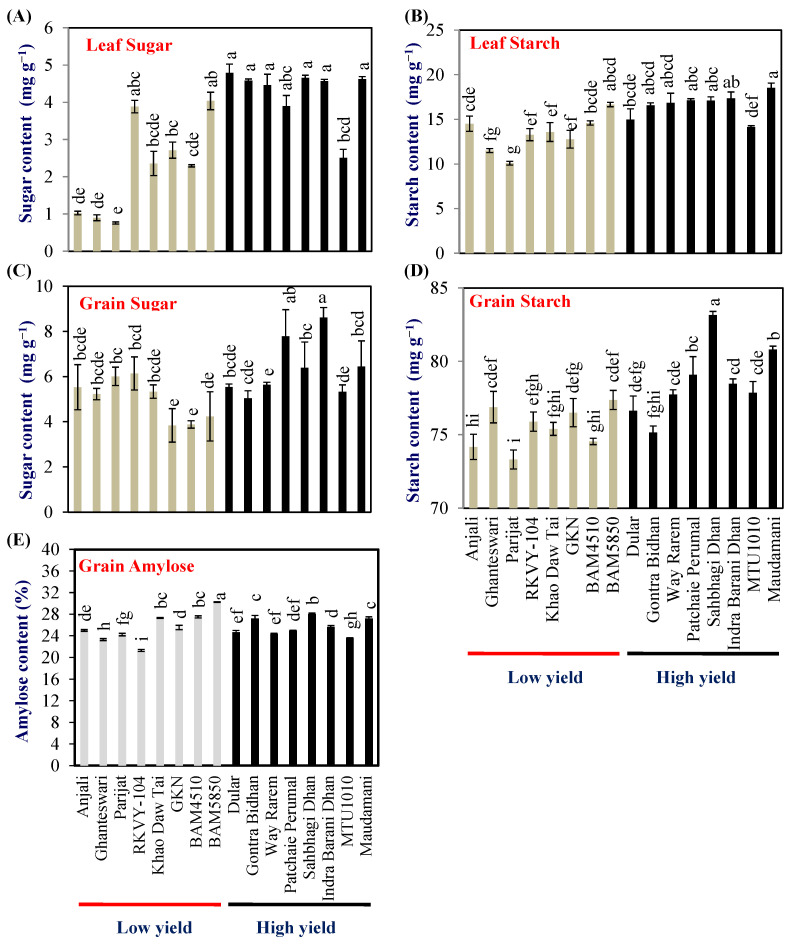
Biochemical analysis of grain carbohydrates (**A**,**B**) flag leaf sugar and starch content, (**C**,**D**) grain sugar and starch content, and (**E**) grain amylose content in the LY and HY groups of rice genotypes. Different letters above the bars indicate statistically significant difference at *p* < 0.05.

Leaf sugar and starch contents indicate the metabolic strength of the source leaf. Hence flag-leaf sugar and starch content were examined at the milking stage of grain development in 2018. The leaf sugar content varied from 0.76 to 4.04 and 2.51 to 4.79 mg g^−1^ dry wt in LY and HY groups, respectively. The mean leaf sugar content of the HY group was 4.26 (±0.2) mg g^−1^ dry wt which was double compared with that of the LY group (2.24 ± 0.1) (Figure 2A). The HY group genotypes Dular, Gontra Bidhan 3, Way Rarem, Sahbhagi Dhan, Indira Barani Dhan-1, and Maudamani accumulated significantly higher leaf sugar content compared with that of all the LY group genotypes (Figure 2A).

The leaf starch content varied from 10.09 to 16.65 and 14.15 to 18.55 mg g^−1^ dry wt in LY and HY groups, respectively. The mean leaf starch content of the HY group was 16.59 (±0.6) mg g^−1^ dry wt, which was about 24% higher compared with that of the LY group (13.37 ± 0.6) (Figure 2B). In the case of leaf starch content, the HY group genotype Maudamani accumulated significantly higher leaf starch content compared with all the LY group genotypes (Figure 2B). 

### 2.4. Grain Sugar and Starch Content

The grain sugar content at harvest varied from 3.8% (BAM5850) to 6.1% (RKVY-104) with a mean of 5.0% in the LY group. The range of grain sugar content for the HY group was 5.0% (Gontra Bidhan) to 8.6% (Indira Barani Dhan-1) and a mean of 6.3%, respectively (Figure 2C). The grain starch content varied from 73.3% (Parijat)–77.3% (BAM4510) and 75.15% (Gontra Bidhan)–83.1% (Sahbhagi Dhan), respectively, in the LY and HY groups (Figure 2D). 

The grain amylose content in the LY genotypes ranged from 21.29% (RKVY104) to 30.25% (BAM4510) while, in the HY group, it ranged from 23.56% (MTU1010) to 28.04% (Sahbhagi Dhan). No clear pattern was observed in grain amylose content between the LY and HY genotypes. One genotype each from the LY and HY groups, i.e., BAM4510 and Sahbhagi Dhan showed significantly higher grain amylose content (Figure 2E).

### 2.5. Expression Profiling of Photosynthesis Regulatory Genes

Expression analysis of genes coding for proteins involved in photosynthesis, viz., ribulose bisphosphate carboxylase small subunit 2 (*RbcS2*), rubisco activase (*RCA*), fructose 1,6 bisphosphatase (*FBPase*), and zeaxanthine epoxidase (*ZEP*), were carried out in flag leaf to assess the source strength when the grain development was at the milky stage in the LY and HY groups of genotypes. The expression level of genes in the LY group rice cv. Parijat served as a calibrator to compare the expression levels of respective genes in other rice genotypes.

**Figure 3:**
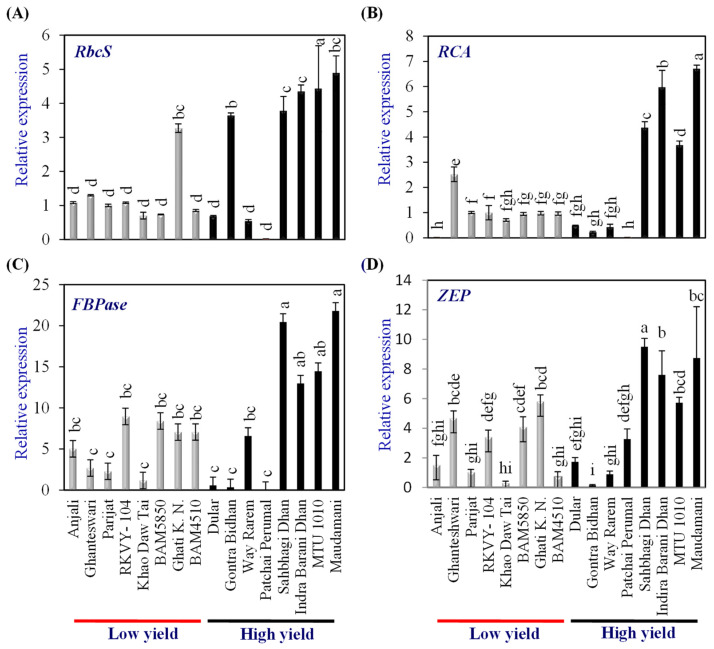
Relative expression of photosynthetic genes in the leaves of contrasting low-yielding (LY) and high-yielding (HY) rice genotypes. *Ribulose bisphosphate carboxylase small subunit* (*RbcS*) (**A**), *Rubisco activase* (*RCA*) (**B**), *Fructose 1,6 bisphosphatase* (*FBPase*) (**C**), and *Zeaxanthine epoxidase* (*ZEP*) (**D**). For relative expression analysis, the expression in Parijat grains was used as a calibrator. Different letters above the bars indicate statistically significant difference at *p* < 0.05.

Among the LY genotypes, only Ghati Karma Nangarhar showed significantly higher expression levels of *RbcS2*, while among the HY group, Gontra Bidhan 3, Sahbhagi Dhan, Indira Barani Dhan-1, MTU 1010, and Maudamani showed significantly higher expression than that of Parijat (Figure 3A). Among the LY genotypes, only Ghanteswari showed significantly higher (2.5 fold) expression levels of *RCA*, while among the HY group, Sahbhagi Dhan, Indira Barani Dhan-1, MTU 1010, and Maudamani showed significantly higher expression than that of Parijat (Figure 3B). The expression levels in these HY genotypes were higher by 3.5 fold to 6.7 fold.

In the case of *FBPase,* LY genotypes RKVY-104, BAM5850, Ghati Karma Nangarhar, and BAM4510 showed significantly higher expression (7.1 to 9.0 fold). However, among the HY group, Sahbhagi Dhan, Indira Barani Dhan-1, MTU 1010, and Maudamani showed *FBPase* expression levels ranging from 13 fold to 21.8 fold, which were significantly higher than that of Parijat as well as that of the LY genotypes, with the highest *FBPase* expression (Figure 3C).

Among the LY genotypes, Ghanteshwari, RKVY- 104, BAM5850, and Ghati Karma Nangarhar showed significantly higher (3.4 fold to 5.81 fold) expression of *ZEP* than that of Parijat (Figure 3D). Among the HY genotypes, Sahbhagi Dhan, Indira Barani Dhan-1, MTU 1010, and Maudamani showed significantly higher expression, which varied from 5.7 fold to 9.51 fold (Figure 3D).

### 2.6. Expression Profiling of Starch-Synthesis Genes in Selected Genotypes 

Sucrose is exported from the source (leaf) to the sink (grain) to provide building blocks and energy to the developing grains. Hence, to understand the synthesis of sucrose in the leaves and grains for the LY and HY rice genotype groups, the expression levels of the *sucrose phosphate synthase* (*SPS*) genes were analyzed. *SPS* genes encode the SPS (EC 2.3.1.14) enzyme, the rate-limiting enzymes involved in sucrose biosynthesis, and catalyze the conversion of fructose-6-phosphate and UDP-glucose into sucrose-6-phosphate.

**Figure 4:**
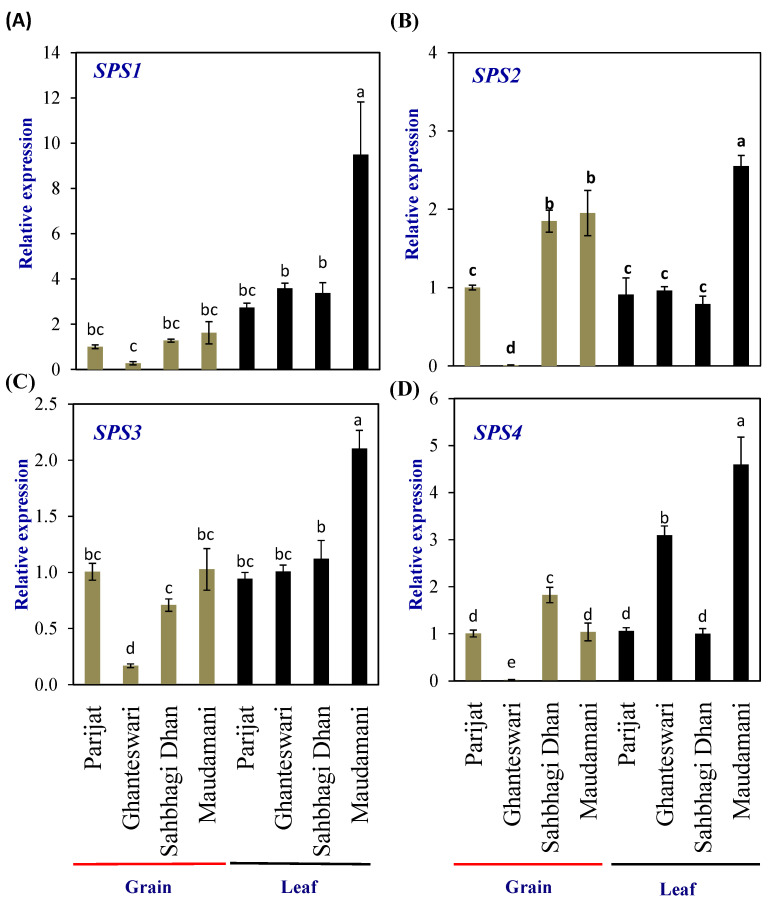
Relative expression of *sucrose phosphate synthase* (*SPS*) genes in selected HY and LY genotypes. (**A**–**D**) Expression of different members of *SPS* family. For relative expression analysis, expression in Parijat grains was used as a calibrator. Different letters above the bars indicate statistically significant difference at *p* < 0.05.

The rice genome encodes five *SPS* genes that can be grouped into four groups, viz., A (*OsSPS8*), B (*OsSPS1*), C (*OsSPS11*), and D (*OsSPS2* and *OsSPS6*) [19]. In the leaf tissues of HY rice cv. Maudamani, all four *SPS* genes showed significantly higher expression than other genotypes. In the grains, no distinct pattern of expression between the LY and HY groups was observed, except for *SPS2,* which showed significantly higher levels of expression in both the HY genotypes compared with the LY genotypes (Figure 4).

**Figure 5:**
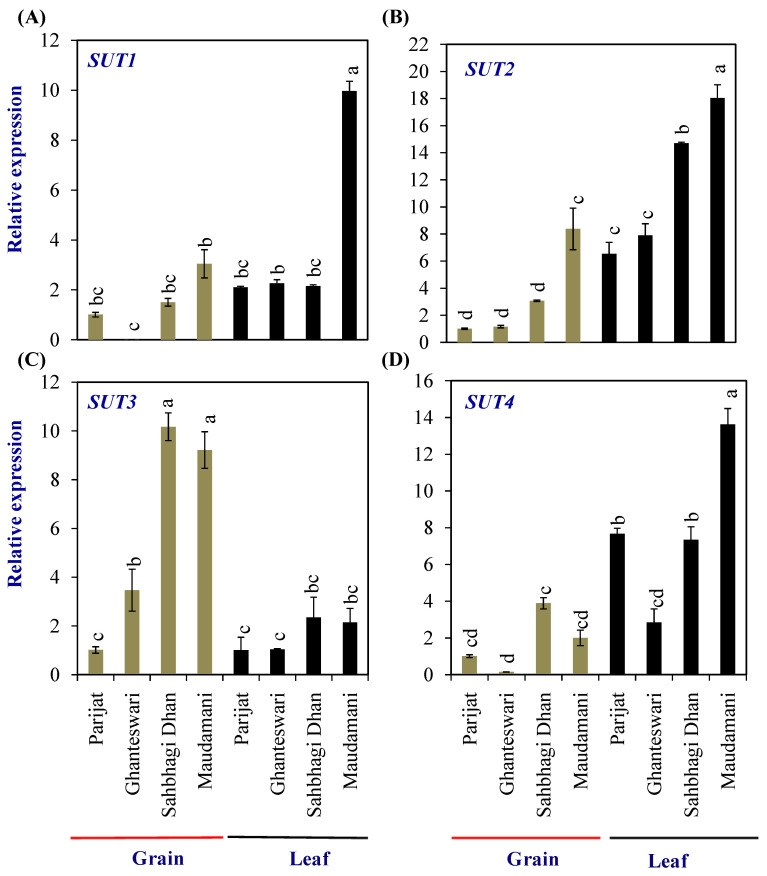
Relative expression of *sucrose transporter* (*SUT*) genes in selected HY and LY genotypes. (**A**–**D**) Expression of different members of *SUT* family. For relative expression analysis, expression in Parijat grains was used as a calibrator. Different letters above the bars indicate statistically significant difference at *p* < 0.05.

Grain-filling initiation in rice requires the import of sucrose into the rice endosperm. The sucrose transporters (SUTs) are sucrose/H+ symporters. In rice, the *SUT* gene family consists of five members, viz., *SUT1*–*SUT5*. Expression of the *SUT1* to *SUT4* genes was analyzed in the leaves and grains of two genotypes each from the LY and HY groups (Figure 5). Among the *SUT*s, the expression levels of *SUT1*, *SUT2,* and *SUT3* in grains, and *SUT1*, *SUT2*, and *SUT4* in leaves were significantly higher in the HY genotype Maudamani compared with Parijat. *SUT3* showed significantly higher levels of expression in the grains of HY genotypes compared with the LY genotypes. Furthermore, *SUT3* expression levels in the grains of HY genotypes were higher than that of the expression level in leaves of both the LY and HY genotypes (Figure 5). 

Adenosine diphosphate glucose pyrophosphorylase (AGPase, EC 2.7.7.27) enzyme is the rate-limiting enzyme for the synthesis of starch, including both amylose and amylopectin. AGPase catalyzes the conversion of glucose-1-phosphate and ATP to ADP-glucose (ADP-Glc) and pyrophosphate. In higher plants, AGPase is a α2β2 heterotetramer consisting of two small subunits (AGPS or SSU = α2) with catalytic activity, and two large subunits (AGPL or LSU = β2) with regulatory properties. To elucidate the molecular mechanism of starch synthesis in a rice leaf sheath, a comprehensive expression analysis was conducted on the gene families encoding starch-synthesis-related enzymes, ADP-glucose pyrophosphorylase (EC 2.7.7.27), starch synthase (EC 2.4.1.21), and branching enzyme (EC 2.4.1.18).

**Figure 6:**
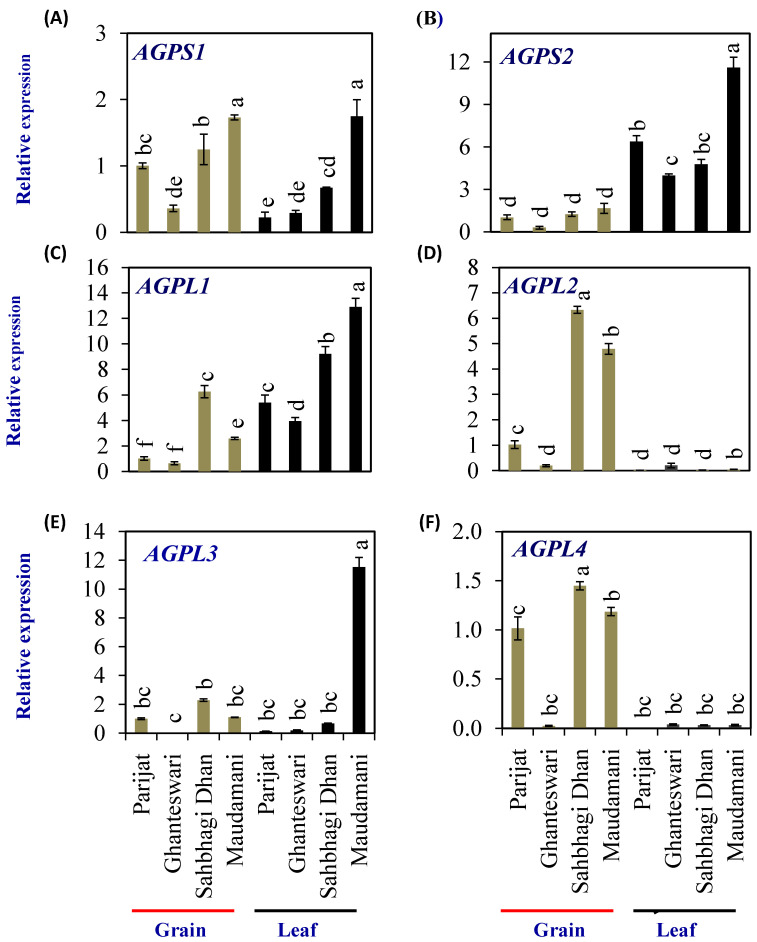
Relative expression of starch-biosynthesis genes in selected HY and LY genotypes. *ADP-glucose pyrophosphorylase small* subunit (*AGPS*)–*AGPS1* (**A**) and *AGPS2* (**B**); and *ADP-glucose pyrophosphorylase large* subunit (*AGPL*)–*AGPL1* (**C**), *AGPL2* (**D**), *AGPL3* (**E**), and *AGPL4* (**F**). For relative expression analysis, expression in Parijat grains was used as a calibrator. Different letters above the bars indicate statistically significant difference at *p* < 0.05.

The rice genome encodes two *AGPS* (*OsAGPS1* and *OsAGPS2*) and four *AGPL* (*OsAGPL1, OsAGPL2, OsAGPL3,* and *OsAGPL4*) genes. The *AGPS1* (Figure 6A) expression levels in both the grain and leaf of the HY genotype Maudamani were significantly higher than that of the LY and HY genotypes. The *AGPS2* gene (Figure 6B) showed significantly higher levels of expression in leaves compared with grains in all genotypes. In grains, Ghanteshwari showed significantly lower *AGPS2* expression compared with Parijat, while it was similar to that of Parijat in HY genotypes. In leaves, *AGPS2* expression was highest (11.6 fold) in the HY genotype Maudamani compared with other genotypes (Figure 6B). 

Among the *AGPL* genes, *AGPL1* expression in leaves was significantly higher in HY rice genotypes Sahbhagi Dhan and Maudhamani compared with LY genotypes (Figure 6C). *AGPL2* expression levels were significantly higher in grains of HY genotypes compared with that of Parijat (Figure 6D). In leaves, *AGPL3* expression was significantly higher in the HY genotype Maudhamani (11.53 fold), and expression was significantly lower in the LY genotype Ghanteswari compared with that of Parijat (Figure 6E). *AGPL4* expression in grains of the LY genotype Ghanteswari was significantly lower while that of HY genotype Sahbhagi Dhan was significantly higher compared with that of the LY genotype Parijat (Figure 6F). 

After the AGPase reaction, the elongation of α-1,4-chains of amylose and amylopectin is catalyzed by granule-bound starch synthase (GBSS) and a soluble starch synthase (SSS) family of enzymes, respectively.

**Figure 7:**
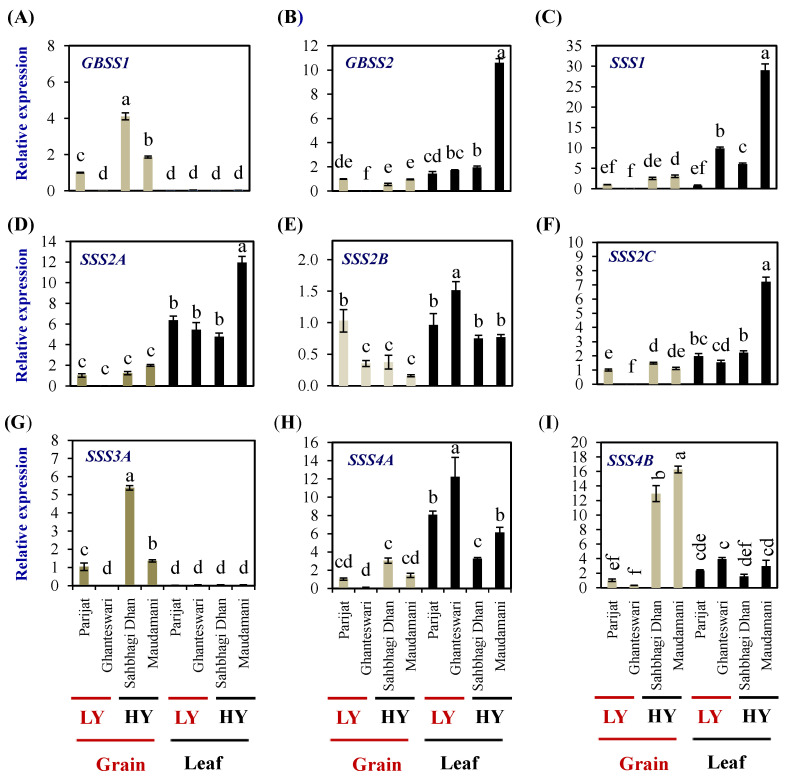
Relative expression of starch biosynthesis genes in the selected HY and LY rice genotypes. *Granule-bound starch synthase GBSS1* (**A**) and *GBSS2* (**B**). *Soluble starch synthase SSS1* (**C**), *SSS2A* (**D**), *SSS2B* (**E**), *SSS2C* (**F**), *SSS3A* (**G**) *SSS4A* (**H**), and *SSS4B* (**I**). For relative expression analysis, expression in Parijat grains was used as a calibrator. Different letters above the bars indicate statistically significant difference at *p* < 0.05.

The rice genome encodes two *GBSS* genes and eight *SSS* genes (one *SSI*, three *SSII*, two *SSIII*, and two *SSIV* genes). In grains, the expression levels of *GBSSI* were significantly higher in both the HY genotypes compared with the LY genotype Parijat, while in leaves, the expression levels were significantly lower than that of the expression in grains of the LY genotype Parijat (Figure 7A). *GBSSII* expression in leaves was higher than that of grains and was highest in the leaves of the HY genotype Maudamani (10.59 fold) (Figure 7B). 

*SSS1* expression was significantly higher in the leaves of the LY genotype Ghanteswari and the HY genotypes Sahbhagi Dhan and Maudhamani (Figure 7C). *SSS1* expression was significantly higher (29 folds) in Maudamani compared to its expression in the rest of the genotypes. 

*SSS2A* expression in grains was significantly lower than that of leaves (Figure 7D). Expression levels of *SSS2B* in grains of Ghanteswari and both of the HY genotypes were significantly lower compared with that of its expression in grains of Parijat, while, in leaves, the expression level was significantly the highest in the leaves of Ghanteshwari (Figure 7E). Expression levels of *SSS2C* in grains of the LY genotype Ghanteshwari were significantly lower compared to that of Parijat, while the expression levels in leaves were significantly higher in leaves of Maudamani (Figure 7F). 

In grains of HY genotypes, the *SSS3A* expression levels were significantly higher in Sahbhagi Dhan (5.38 fold), while, in leaves, the expression levels were on par among genotypes and significantly lower than that of grains of Parijat (Figure 7G). The expression levels of *SSS4A* were significantly higher in leaves compared with that of grains, and the highest expression was observed in leaves of the LY genotypes Ghanteswari (12 fold) and Parijat (8 fold) (Figure 7H). The *SSS4B* expression levels were significantly higher in grains of the HY genotypes Sahbhagi Dhan (12 fold) and Maudhamani (16 fold) than that of the LY genotypes (Figure 7I).

### 2.7. SNP-Based Allele Mining and Generation of Dendrogram

The analyses of all 22 genes were done for probable amino acid changes using the genotype data from the SNP Chip. Among them, four genes, namely soluble starch synthase 3A (*SSS3A*, Os08g09230), soluble starch synthase 4A (*SSS4A*, Os01g52250), soluble starch synthase 1 (*SSS1*, Os06g06560), and sucrose transporter 5 (*SUT5*, Os02g36700) were found to have functional SNPs (Table S5). We could not find any SNP which showed a direct correlation with contrasting genotypes. However, the soluble starch synthase 3A gene (Os08g09230) recorded the highest number of functional SNPs (Table S5).

**Figure 8:**
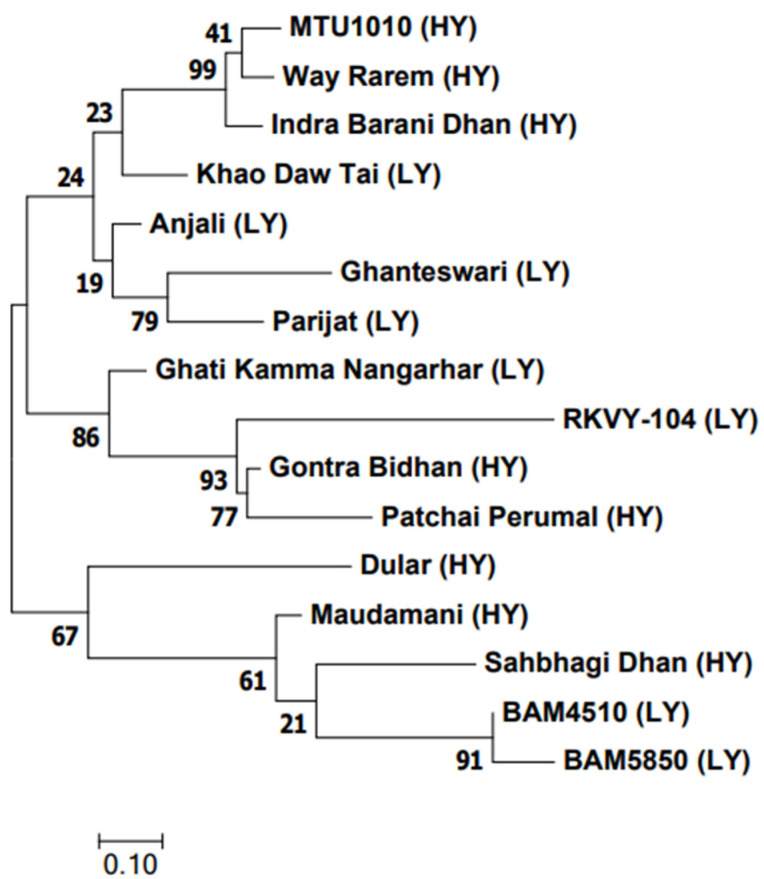
Dendrogram of 16 contrasting yield traits of rice genotypes.

The genetic relationship among the 16 genotypes using the 50 K SNP data categorized them into four groups (Figure 8). There was no distinct grouping between the LY and HY genotypes. Yet, in group I, three HY genotypes were clustered together in one subgroup, while the LY genotype Khao Daw Tai made a separate branch. The second group showed a cluster of three LY genotypes. The third and fourth groups consisted of HY and LY genotypes. 

## 3. Discussion

In this study, an attempt was made to analyze the genotypic differences in biomass accumulation and grain yield based on the component physiological, biochemical, and molecular traits of source and sink activity. Physiological traits are the key attributes of rice genotypes that determine yield potential. Crop yield is influenced by both genotype and environment and is a major determinant of source-to-sink potential. We found that the mean performance of the HY genotypes was higher in comparison to the LY genotypes for relevant physiological traits. 

Grain yield is a function of the total biomass produced and the fraction of biomass partitioned (harvest index) for grain production [20]. This study clearly showed that the HY group of genotypes produced significantly higher biomass (1.7 fold) than that of the LY group, with only marginal differences in HI between these groups.

Biomass accumulation per unit of land area depends upon incident solar radiation received, the total amount of PAR intercepted, and the radiation-use efficiency of crops [7]. In this study, the total crop duration, which determines the total amount of radiation received, differs only by about 6 days between the LY and HY genotypes (~6% higher in HY genotypes). However, the mean biomass accumulation over two years in the HY genotype group is about 67% higher than that of the LY genotype group. This suggests that the biomass accumulation differences between LY and HY groups may be mainly due to the differences between these groups of genotypes in the total amount of PAR intercepted and radiation-use efficiency.

Earlier studies showed that enhancing radiation interception by enhanced leaf area and architecture can increase the RUE and yield [20,21,22]. In the rice leaf-area index (LAI) 95% interception is about 6.7 and 9.7 in semidwarf and dwarf rice varieties, and canopy photosynthesis increases at a higher rate until an LAI of 9 [23]. In this study, HY genotypes had about 66% higher leaf area per plant compared with that of the LY group. The calculated LAI, based on leaf area (LA) per plant (33 plants per m^2^), was 4.51 (±0.42) and 7.97 (±0.82). Thus, it clearly showed that a higher leaf area per plant in rice is critical for higher biomass accumulation.

Further, the source strength was analyzed in terms of leaf chlorophyll content, expression of genes for photosynthesis, and its partitioning. Total chlorophyll content and photosynthetic rate were significantly higher in the HY group compared with the LY group. Although the relationship between net photosynthesis measured by IRGA on a single leaf with biomass in rice and other crops is not perfect [24], in several cases an increase in the photosynthetic rate at a leaf level has been shown to be associated with an increase in biomass and grain yield [7,25].

As evident from this study, the marginal advantage of the HY group in the photosynthetic rate at a leaf level gets amplified due to the significantly higher flag-leaf area and total leaf area per plant. This leads to potentially a higher canopy photosynthesis in the HY group compared with the LY group. 

Among other source traits, such as leaf sugar and starch content, the sucrose content in grains of HY genotypes was, in general, high (except MTU1010 and Patchai Perumal) compared with LY genotypes. To further understand photosynthesis and source activity, a gene-expression analysis was carried out. Rubisco activase (RCA) activates Rubisco by removing inhibitory sugar phosphates from the active site of Rubisco, thereby enhancing the photosynthesis rate [26,27]. Fructose-1,6-bisphosphatase (FBPase) catalyzes the synthesis of fructose-6-phosphate, which is partitioned into the synthesis of sucrose and the regeneration of RuBP, and thus a key enzyme in the Calvin cycle. Overexpression of FBPase has also been shown to enhance photosynthesis and biomass accumulation [28,29]. In this study, four of the HY genotypes exhibited very high levels of *RCA*, as well as *FBPase* expression and, thus, potentially contributed to photosynthesis and biomass production in these genotypes.

Zeaxanthin epoxidase (ZEP) is a key enzyme in the xanthophyll cycle, and its overexpression has been shown to enhance low light tolerance in plants [30]. In this study, the HY rice genotypes Sahbhagi Dhan, Indra Barani Dhan, and Maudamani showed significantly higher *ZEP* expression than all other genotypes. Higher expression of *ZEP* and other genes involved in the xanthophyll cycle in the lower leaves of rice may enhance low light tolerance and photosynthesis in rice.

In terms of sink strength, rice crop yield depends upon grain number per unit area and the test weight of grain. This study showed that grain number per panicle, and thus grain number per plant, was a major contributing factor for grain yield in the HY group, while test weight was similar between the HY and LY groups. Similar results have been obtained in rice in several studies [31]. Gene-expression analysis and carbohydrate analysis could not clearly establish the relationship between high yield and source–sink traits. 

In conclusion, this study analysed source and sink traits to understand the grain yield. The significant finding of this study is that canopy photosynthesis is the major contributor to high biomass accumulation rather than unit leaf photosynthesis. As evident from the results of this study, high- and low-yielding genotypes differ only marginally in the leaf photosynthetic rate, but high-yielding varieties produce a 68% higher leaf area compared to the low-yielding genotypes, and, thus, canopy photosynthesis contributes to high grain yield.

## 4. Materials and Methods

### 4.1. Plant Material, Experimental Design, and Crop Management

Rice minicore germplasm [32], breeding lines, germplasms, and released varieties of a total of 188 genotypes were evaluated in field conditions at the experimental farm of the ICAR-Indian Agricultural Research Institute (ICAR-IARI), New Delhi, India (latitude: 28°38′23″ North, longitude: 77°09′27″ East, altitude: 228.61 m above MSL) during the *kharif* season (June–November) in 2016 and 2017. The experiment was performed in a randomized complete block design (RCBD) with three replications. Rice nurseries were sown on 14 June 2016 and 13 June 2017, and transplanted on 14 July 2016 and 13 July 2017, respectively. Twenty-five-day-old seedlings were manually transplanted in a puddled field at a spacing of 20 × 15 cm with two square meters (2 m^2^) of area per replicate. Irrigation and agronomic practices were followed as per recommended practices. Irrigation was adjusted according to precipitation and the meteorological data were retrieved from an observatory located adjacent to the experimental field. The detailed analysis of the yield components of 188 genotypes will be published separately. Based on the two-year data, contrasting genotypes (8 high yielding and 8 low yielding) were selected and presented here with further analysis of the source–sink relationships. The details of the low-yield (LY) and high-yield (HY) groups of genotypes selected are given in Table 1. Observations on phenology, viz., days to anthesis and days to physiological maturity, were recorded.

### 4.2. Physiological Parameters

The photosynthetic gas exchange of flag leaf at anthesis was measured using the LI-6400XT portable photosynthesis system (LI-COR Inc., Lincoln, NE, USA) at ambient CO_2_ partial pressure, with the flow rate of 500 mL s^−1^ and fixed light intensity of 1200 μmol m^−2^ s^−1^. Chlorophyll was extracted using DMSO and quantified spectrophotometrically at A_645_ and A_663_ nm with a dual-beam UV-VIS spectrophotometer (UV-VIS 35 model; Beckman Beckman Coulter Life Sciences, Indianapolis, IN, USA). Total chlorophyll content was estimated following the method of Arnon and Whatley, 1949 [33]. The leaf area of the flag leaf was calculated by measuring the length and width of the leaf (leaf area = length × width × 0.75), while the whole plant leaf area was measured using a bench-top leaf-area meter (Li3000C model, LiCOR Inc, Lincoln, NE, USA). 

Sink-related traits, viz., panicle number per plant, grain number per plant, and grain number per panicle, were recorded based on nine plants (three plants per plot). The test weight was measured by weighing 1000 grains. Observations on straw weight per plant and grain yield per plant were recorded at maturity. The straw was dried in an oven at 70 °C for 72 h, and the weight was recorded using an electronic balance. Biomass was calculated as the sum of straw weight and grain yield. The harvest index was calculated as % of grain weight to the total biomass.

### 4.3. Biochemical Analysis

Sixteen contrasting genotypes were used for biochemical analysis. The flag leaf and developing grain at the milking stage were collected, flash-frozen in liquid nitrogen, and stored at −80 °C for biochemical and molecular studies. Grains harvested at maturity were used for the analysis of carbohydrates (starch, sugar, and amylose).

#### Estimation of Starch, Sugar, and Amylose Content

Mature grains harvested at maturity were air dried to about 12% moisture content. Rice grains were dehusked and polished prior to biochemical analysis. It was finely ground in a pestle and mortar, and 50 mg of the sample were taken for analysis. Soluble sugars were extracted in 10 mL of 80% ethanol in a boiling water bath thrice. The extract was pipetted to a fresh tube and was made up to 50 mL with distilled water and mixed thoroughly. The starchy material was hydrolysed in 10 mL of 1 N HCl for 2 h in a boiling water bath. The extract was made up to 50 mL and mixed thoroughly. The cellular debris in grains was allowed to settle down by incubating at room temperature for 1 h. One mL of alcohol extract and the acid-digested samples were used for the analysis of sugar content by the Anthrone method [34]. The absorbance of both the glucose standard and samples was taken at 620 nm. Based on the standard curve, the total glucose content in the digested sample was estimated. The conversion factor of glucose to starch was taken as 0.9.

For the estimation of amylose content, 100 mg of rice-grain powder was used. In test tubes containing the sample, digestion was set up with 1 mL of ethanol (95%) and 9 mL of 1 N NaOH. A slow and thorough shaking was given to all tubes and were transferred to a water bath at 100 °C for 15 min. Then, the samples were cooled to room temperature and the digested samples were transferred to a volumetric flask and the volume was made up to 100 mL. The colorimetry was done by the KI method [35]. Five mL of the sample were transferred to a fresh volumetric flask, to which 1 mL of acetic acid and 2 mL of KI + I solution were added. The volume was made up to 100 mL with distilled water and incubated in the dark for 20 min until a uniform blue colour was obtained. The absorbance at 620 nm was recorded using a spectrophotometer. Based on the standard curve, the amylose content in the sample was estimated. 

### 4.4. RNA Extraction and Gene-Expression Analysis

The total RNA was isolated from the flag leaf and developing grain at the milky stage using the Trizol method [36]. It was treated with DNaseI (TURBO DNA-free™ Kit, Thermo Fischer Scientific, Waltham, MA, USA) to remove genomic DNA contamination, if any. The cDNA synthesis was done using the PrimeScript 1st strand cDNA Synthesis Kit (TaKaRaBio Inc., Shiga, Japan). The qRT–PCR reaction was prepared using KAPA SYBR^®^ FAST qPCR Master Mix (Merck, Darmstadt, Germany) with Ubiquitin as an internal reference. The gene sequences for designing the primers were selected from the RGAP (rice.plantbiology.msu.edu) database. Three replicates were used for each sample. A list of primers used in the qRT–PCR analysis of genes for starch synthesis, sucrose synthesis, and photosynthesis is given in Table S6. The relative expression levels of genes were calculated based on the comparative Ct method using the 2^ΔΔCt^ method [37].

### 4.5. Allele Mining of Candidate Genes

Allele mining was performed with the 50 K SNP chip [38] genotype data generated for these genotypes in our lab. The method involved three basic steps, viz. finding the intron and exon of the gene sequence, identifying the position of the SNP in the gene sequence, and demarcation of the codon that the SNP is spanning. For finding the intron and exon of the gene sequence, the CDS sequence was given as an input in the OryGenes DBdatabase [39]. The corresponding locus ID of the candidate gene was selected and the colour-coded sequence demarcating the UTR/intron/exon was retrieved. The probe sequence was demarcated in the colour-coded sequence. To identify the location of the probe and point of SNP in the gene sequence, the find and search option in MS Office Word was employed. If the SNP was found to be exonic, the amino acid sequence, along with the corresponding codon, was deduced from the CDS sequence. Here, the Expasy [40] translate tool with the output as compact format was used. The probe sequence and the position of the SNP were demarcated in the translated sequence output. From this, the exact triplet codon containing the SNP was highlighted. The change in amino acid due to nonsynonymous SNP or silent mutation was identified using a eukaryotic codon sequence chart. 

To construct the dendrogram among the genotypes, the results from the 50 K SNP chip data were used. The allelic variations of all the 50 K probes spanning the 16 genotypes were collected from the 50 K SNP analysis. The data were analysed in MEGA 7.0 software [41]. The input data was saved as an “*.aln” file and used for the construction of the dendrogram. The genetic relationship was inferred by using the maximum likelihood method based on the Tamura–Nei model [42]. The genetic relationship was generated by the maximum likelihood method with 1000 bootstrap replicates [43]. Allele mining of sixteen (8 LY and 8 HY) contrasting rice genotypes and their alternative codons with Locus ID is given in Table S5.

### 4.6. Statistical Analysis

All the statistical analyses were performed using R statistical analysis software (https://www.r-project.org/, accessed on 15 April 2022) [44] using the agricolae package [45]. MEGA 7.0 (Molecular Evolutionary Genetic Analysis) software was used for conducting manual sequence alignment and phylogenetic trees.

## 5. Conclusions

In conclusion, this study clearly showed that the development of high biomass genotypes is critical for higher grain yield in rice. This can be potentially achieved by enhancing the leaf area per plant with more electrophilic leaves, to achieve higher interception of PAR, and enhancing the photosynthetic rate at the leaf and canopy level. From the source strength side, enhancing grain number per panicle, and panicle number per unit area will be important for achieving higher grain yield. Our findings have potential implications for improving the source–sink balance in rice for developing rice genotypes with a higher yield.

## Figures and Tables

**Table 1:** Rice genotypes with contrasting yield and biomass.

Sl No.	Genotypes	Details of the Genotypes/Parentage etc.
	**Low Yielding (LY)**	
1	Anjali	Parentage: PR-19-2 X RR-149-1129, released in 2002 by the National Rice Research Institute (NRRI), Cuttack, early maturing (90–95 days), semi-tall (85–100 cm), suitable for rainfed, direct-seeded conditions, grains: short bold, white, moderately tolerant to drought, resistant to brown spot, moderately resistant to leaf blast, yield: 3 t/ha under direct seeded, and 4 t/ha under transplanted conditions.
2	Ghanteswari	Parentage—IR-2061-628- 1-6-4-3 x N-2-2, released in 1994, early maturing (90–95 days), dwarf (85 cm), resistant to blast, neck blast, yield: 35 Q/ha. High silicon uptake ability.
3	Parijat	Parentage—TKM-6xT(N)1, released in 1985, duration 95–100 days, dwarf (85 cm), medium slender grains, moderately resistant to sheath blight, blast, leaf blight and helminthosporium leaf spot, yield: 30–40 q/ha.
4	RKVY-104	Duration (105 days), medium plant height,
5	Khao Daw Tai	Duration (105 days)
6	Ghati Kamma Nangarhar	Duration (110 days), medium plant height.
7	BAM4510	IC-6294, germplasm line from Northeast India
8	BAM5850	IC38224, germplasm line from Northeast India
	**High Yielding (HY)**	
9	Dular	It is an upland-adapted, drought-tolerant traditional genotype from India and has shown consistent performance in drought screening at IRRI. It is early maturing.
10	Gontra Bidhan 3	Duration—110–115 days, plant height—95–100 cm, short medium bold grain type, average yield—6t/ha, suitable for medium to medium upland.
11	Way Rarem	It is an Indica variety from Indonesia. Medium plant height and higher chlorophyll contents.
12	Patchai Perumal	It is a water-deficit stress-tolerant variety.Tall plant height, mostly cultivated in Kerala, India and Sri Lanka.
13	Sahbhagi Dhan	Dwarf statured (90–100 cm), drought-tolerant variety suitable for upland, rainfed direct seeded as well as transplanted conditions. It bears golden-husked long bold grains and yields 3.8–4.5 t/ha. It is resistant to leaf blast and moderately resistant to brown spot, sheath rot, stem borer, leaf folder, etc. It is cultivated in Jharkhand and Odisha. Released by NRRI, Cuttack in 2009.
14	Indira Barani Dhan-1	Parentage—Swarna x IR 42253, maturity—111–115 days, medium slender grain, suitable for rainfed shallow lowland ecosystem of Chhattisgarh plains and adapted to aerobic conditions, average yield 40–45 q/ha.
15	MTU 1010	An elite, high-yielding, short-duration, widely cultivated megavariety, possessing long slender grain type, was developed and released by Acharya NG Ranga Agricultural University (ANGRAU), Guntur, Andhra P.
16	Maudamani	Parentage/Pedigree: Dandi/Naveen/Dandi. It is also known as CR DHAN 307. Released by ICAR-National Rice Research Institute, Cuttack in 2014.

## Data Availability

Data is contained within the article and supplementary material.

## Supplementary Materials

The following supporting information can be downloaded at: https://www.mdpi.com/article/10.3390/plants13010062/s1, Table S1. ANOVA of grain yield, biomass and HI in Low yielding (LY) and High yielding (HY) rice genotypes. Table S2. ANOVA of two (2) phenological traits- anthesis days and maturity days in 2016 and 2017, respectively. Table S3. ANOVA of yield components in Low yielding (LY) and High yielding (HY) rice genotypes. Table S4. ANOVA of source traits in Low yielding (LY) and High yielding (HY) rice genotypes. Table S5. Allele mining of sixteen (8-LY and 8-HY) contrasting rice genotypes. Table S6. List of genes of photosynthetic, starch synthesis and sucrose transport and used for study of expression profile.
