# Supplementary material for: Physiological Analysis of Source–Sink Relationship in Rice Genotypes with Contrasting Grain Yields
Source: Plants (Basel). 2023 Dec 23;13(1):62. doi: 10.3390/plants13010062 (PMC10780537; doi:10.3390/plants13010062)
Supplement: Supplementary file 1 [file plants-13-00062-s001.zip › plants-2559709-supplementary.pdf]

Article

Physiological analysis of source-sink relationship in rice genotypes with contrasting grain yield

Chandrapal Vishwakarma[1], Gopinathan Kumar Krishna[2], Riti Thapar Kapoor[3], Komal Mathur[3], Monika Dalal[4], Nagendra Kumar Singh[4], Trilochan Mohapatra[5], Viswanathan Chinnusamy[1*]

Table S1.

ANOVA of grain yield, biomass and HI in Low yielding (LY) and High yielding (HY) rice genotypes.

Grain yield per plant (GY/plant (g)), Biomass per plant (BM/plant (g)) and Harvest index % (HI%); [*Ghati Kamma Nangarhar (Ghati K N)].

Traits	GY/plant (g)		BM/plant (g)		HI (%)	
**Genotypes**	**Mean + SE**	**Mean + SE**	**Mean + SE**	**Mean + SE**	**Mean + SE**	**Mean + SE**
**Year**	**2016**	**2017**	**2016**	**2017**	**2016**	**2017**
**Low Yield**						
Anjali	15.9±1.4	10.0±0.3	30.8±4.4	20.3±1.2	53.2±5.9	49.6±4.1
Ghanteswari	9.5±1.0	10.9±2.5	21.6±3.7	20.2±3.5	45.2±4.0	52.9±5.0
Parijat	15.6±2.7	17.4±0.8	30.4±4.2	37.4±2.8	51.7±8.2	47.0±4.5
RKVY-104	8.7±0.3	14.9±0.7	28.0±2.9	30.1±4.5	32.1±3.7	51.0±5.7
Khao Daw Tai	15.0±2.4	15.6±1.1	33.1±2.5	33.1±1.8	44.9±5.7	46.8±1.1
Ghati K N*	9.5±1.0	12.7±1.0	25.9±2.8	28.6±0.7	37.2±4.8	44.6±4.4
BAM4510	17.2±3.1	13.5±3.2	37.5±3.9	31.2±1.7	45.2±3.8	44.7±12.2
BAM5850	9.8±2.2	18.6±0.6	34.4±2.6	39.0±2.0	27.9±4.8	47.8±3.1
**Range**	8.7±0.3-17.2±3.1	10.0±0.3-18.6±3.2	21.6±2.5-37.5±4.4	20.2±0.7-39.0±4.5	27.9±3.7-53.2±8.2	44.6±1.1-52.9±12.2
**Mean**	**12.6±1.8**	**14.2±1.3**	**30.2±3.4**	**30.0±2.3**	**42.2±5.1**	**48.1±5.0**
**High Yield**						
Dular	30.6±1.5	23.0±2.8	57.4±7.6	49.1±6.6	54.7±5.1	47.0±1.5
Gotrabi Dhan	31.2±5.4	22.1±0.8	63.6±12.7	47.4±5.5	49.9±2.0	47.5±4.2
Way Rarem	19.8±0.6	16.9±0.9	42.2±2.3	37.0±1.9	47.0±1.6	45.8±1.7
Patchaiperumal	28.3±2.0	26.0±2.0	55.9±7.7	50.6±3.3	51.7±3.9	51.3±2.0
Sahbhagi Dhan	20.8±1.3	25.6±2.4	42.2±3.1	45.4±3.0	49.3±0.5	56.3±1.6
Indrabaruni Dhan	26.1±2.7	33.7±1.9	50.8±6.5	59.2±4.2	51.8±1.6	57.1±0.9
MTU1010	24.3±2.0	29.8±6.2	50.6±4.6	50.0±5.0	48.2±1.1	58.5±6.2

Maudamani	23.5±0.4	27.1±0.4	59.1±8.0	53.0±2.5	40.9±4.4	51.4±2.9
**Range**	19.8±0.4-31.2±5.4	16.9±0.4-33.7±6.2	42.2±2.3-63.6±12.7	37.0±1.9-59.2±6.6	40.9±0.5-54.7±5.1	45.8±0.9-58.5±6.2
**Mean**	**25.6±2.0**	**25.5±2.2**	**52.7±6.6**	**49.0±4.0**	**49.2±2.5**	**51.9±2.6**
**C.D.**	6.591	6.653	16.702	9.437	12.646	NS
**C.V.**	20.577	19.995	24.042	14.263	16.523	16.416

Table S2.

ANOVA of two (2) phenological traits- anthesis days and maturity days in 2016 and 2017, Respectively. [*Ghati Kamma Nangarhar (Ghati K N)].

**Traits**	**Days to Anthesis**		**Days to Maturity**	
	**Mean + SE**	**Mean + SE**	**Mean + SE**	**Mean + SE**
**Year**	**2016**	**2017**	**2016**	**2017**
**Low Yield**				
Anjali	67.3±1.2	66.3±0.7	91.0±0.6	90.0±0.0
Ghanteswari	74.0±0.6	76.0±2.5	104.0±0.6	104.3±0.3
Parijat	81.0±0.6	83.7±0.9	104.3±0.7	107.3±0.3
RKVY-104	83.3±1.2	85.3±0.9	106.7±0.7	109.3±0.7
Khao Daw Tai	86.7±1.8	86.7±1.3	108.3±1.5	111.0±0.0
Ghati K N*	88.0±1.2	89.3±1.2	114.0±1.2	114.7±0.7
BAM4510	90.0±2.0	93.7±0.9	113.7±0.9	115.3±0.7
BAM5850	88.7±0.9	92.0±1.2	114.0±1.2	117.0±2.0
**Range**	67.3±1.2 - 90.0±2.0	66.3±0.7 - 93.7±0.9	91.0±0.6 - 114.0±1.2	90.0±0.0 - 117.0±2.0
**Mean**	**82.4±1.17**	**84.1±1.19**	**107.0±0.89**	**109.0±0.64**
**High Yield**				
Dular	79.7±0.9	82.0±0.6	103.3±0.7	104.7±0.7
Gontra Bidhan	81.7±0.9	81.0±0.6	104.0±0.6	105.0±0.0
Way Rarem	92.7±1.2	93.7±1.2	117.7±0.3	116.7±1.2
Patchai Perumal	85.0±2.1	90.7±2.3	112.0±1.2	115.0±0.6
Sahbhagi Dhan	84.7±1.2	87.7±2.4	110.7±0.7	112.7±0.7
Indra Barani Dhan	88.0±1.2	92.0±1.7	112.0±1.2	114.7±1.8
MTU1010	87.7±2.6	91.0±0.6	112.0±1.2	114.7±0.3
Maudamani	111.0±0.6	113.3±0.9	135.7±1.2	136.0±1.0
**Range**	79.7±0.9 - 111.0±0.6	81.0±0.6 - 113.3±0.9	103.3±0.7 - 135.7±1.2	104.7±0.7 - 136.0±1.0

**Mean**	**88.8±1.32**	**91.4±1.29**	**113.4±0.86**	**114.9±0.78**
C.D.	3.55	3.17	2.64	2.30
C.V.	2.47	2.16	1.43	1.23

Table S3.

ANOVA of yield components in Low yielding (LY) and High yielding (HY) rice genotypes.
Panicle number per plant (PN/plant), Grain number per plant (GN/plant), Grain number per panicle (GN/panicle), 1000 grain weight (Test wt (g)); [*Ghati Kamma Nangarhar (Ghati K N)].

Traits	PN/plant		GN/plant		GN/panicle		Test wt (g)	
Genotypes	Mean + SE	Mean + SE	Mean + SE	Mean + SE	Mean + SE	Mean + SE	Mean + SE	Mean + SE
Year	2016	2017	2016	2017	2016	2017	2016	2017
**Low Yield**								
Anjali	8.3±1.2	9.0±0.6	678.3±79.9	410.7±13.2	83.0±11.5	45.5±2.6	23.6±1.0	24.3±0.3
Ghanteswari	7.3±1.2	8.0±1.0	453.0±38.1	585.0±108.5	67.0±9.6	75.6±15.5	21.0±0.6	18.3±0.9
Parijat	7.3±1.2	9.3±0.9	761.2±150.7	815.3±71.0	102.8±8.7	90.4±8.8	20.8±0.6	21.5±1.1
RKVY-104	10.3±0.3	7.3±0.9	376.1±0.3	597.4±37.0	52.7±15.7	80.9±10.9	23.3±0.8	25.1±1.5
Khao Daw Tai	9.7±0.7	10.3±1.2	690.0±121.8	704.1±63.2	90.9±5.3	69.8±6.0	21.9±0.9	22.2±0.5
Ghati K N*	7.3±0.9	8.7±0.3	482.5±19.3	611.5±29.6	67.7±10.9	69.8±1.1	19.6±1.6	20.9±2.1
BAM4510	10.3±2.3	8.0±1.2	883.3±131.9	608.0±147.2	90.8±7.3	80.0±26.8	19.3±0.6	22.3±0.1
BAM5850	9.0±1.0	10.0±1.5	482.1±115.1	829.7±44.1	53.2±9.1	88.0±11.3	20.5±0.8	22.4±0.6
**Range**	7.3±0.3-<br>10.3±2.3	7.3±0.3-<br>10.3±1.5	376.1±0.3-<br>883.3±150.7	410.7±13.2-<br>829.7±147.2	36.0±1.0-<br>102.8±16.7	45.5±1.1-<br>90.4±26.8	19.3±0.6-<br>23.6±1.6	18.3±0.1-<br>25.1±2.1
**Mean**	**8.7±1.1**	**8.8±0.9**	**600.8±82.1**	**645.2±64.2**	**76.02±9.77**	**75.0±10.4**	**21.2±0.9**	**22.1±0.9**
**High Yield**								
Dular	9.0±0.6	8.3±0.9	1092.9±61.1	862.8±105.4	123.0±2.5	101.5±4.2	28.0±0.2	26.7±0.6
Gotrabi Dhan	9.3±1.2	8.0±0.6	1443.3±296.5	1010.1±37.6	149.4±15.0	129.2±11.1	22.0±0.8	21.9±0.4
Way Rarem	4.7±0.3	9.7±0.3	811.3±30.8	761.6±44.3	171.5±10.8	79.3±7.6	24.4±0.4	22.3±0.5
Patchaiperumal	10.7±0.3	9.7±0.3	1201.5±70.2	1034.5±67.3	112.5±4.0	107.1±7.4	23.6±1.0	25.1±0.3
Sahbhagi Dhan	7.3±0.9	6.0±0.6	943.8±125.7	1065.0±120.8	126.4±6.3	176.4±9.3	22.4±1.4	24.2±0.7
Indrabaruni Dhan	9.3±0.9	10.0±0.6	1231.4±128.7	1589.7±131.5	132.8±12.9	155.3±16.9	21.2±0.7	21.3±0.9
MTU1010	9.3±1.2	7.3±0.3	1018.7±76.3	1205.8±221.3	114.8±17.1	168.3±33.4	23.9±0.4	24.5±0.7
Maudamani	6.3±0.9	6.0±0.6	1032.5±31.2	1188.4±33.8	160.8±13.8	201.2±16.8	22.7±0.3	22.8±0.4
**Range**	4.7±0.3-<br>10.7±1.2	6.0±0.3-<br>10.0±0.9	811.3±30.8-<br>1443.3±296.5	761.6±33.8-<br>1589.7±221.3	112.5±2.5-<br>171.5±17.1	79.3±4.2-<br>201.2±33.4	21.2±0.2-<br>28.0±1.4	21.3±0.3-<br>26.7±0.9
**Mean**	**8.2±0.8**	**8.1±0.5**	**1096.9±102.6**	**1089.7±95.2**	**136.4±10.3**	**139.8±13.3**	**23.5±0.7**	**23.6±0.6**
**C.D.**	3.048	2.4	336.737	284.019	32.515	42.229	2.378	2.344
**C.V.**	21.456	16.892	23.675	19.54	18.271	23.465	6.34	6.123

Table S4.

ANOVA of source traits in Low yielding (LY) and High yielding (HY) rice genotypes.

Total Chlorophyll (mg/g dry wt), Flag leaf thickness (mm), Leaf area (cm^2), Leaf area ratio (LAR) cm^{-2} g^{-1}, Photosynthetic rate (Pn) (µmol CO_2 m^{-2} s^{-1}), Water Use Efficiency (WUE) (µmole/mole), [*Ghati Kamma Nangarhar (Ghati K N)], NA-Not Available

**Traits**	**Total Chlorophyll**	**Flag Leaf Thickness (mm)**	**Leaf area (cm^2) (86 days)**	**Per Flag leaf area (/FLA)**	**Photosynthetic rate**	**Water use efficiency**
**Genotypes**	**Mean + SE**	**Mean + SE**	**Mean + SE**	**Mean + SE**	**Mean + SE**	**Mean + SE**
**Low Yield**						
Anjali	7.5±0.23	0.276±0.01	1226.11±86.9	NA	26.0±0.06	0.17±0.00
Ghanteswari	8.2±0.33	0.253±0.00	1015.35±87.7	47.1±0.8	22.6±0.62	0.13±0.00
Parijat	7.6±0.22	0.326±0.02	1341.50±49.4	46.5±2.6	23.4±0.43	0.15±0.00
RKVY-104	8.6±0.16	0.535±0.02	1338.73±49.8	45.3±4.9	24.4±0.72	0.21±0.00
Khao Daw Tai	8.0±0.29	0.316±0.01	1192.66±90.0	59.0±7.8	25.7±0.65	0.22±0.00
Ghati K N*	6.9±0.31	0.343±0.02	1187.04±27.4	70.7±6.4	19.3±1.18	0.23±0.05
BAM4510	5.5±0.32	0.286±0.00	1292.97±86.5	NA	18.0±0.15	0.24±0.04
BAM5850	5.8±0.13	0.347±0.03	2199.56±49.2	56.3±4.9	25.7±0.29	0.25.±0.00
**Range**	5.5-8.6	0.253-0.535	1015.36-2199.56	45.3- 70.7	18.0 -26.0	0.13 -0.25
**Mean**	**7.3±0.25**	**0.34±0.02**	**1349.23±65.86**	**54.1±4.6**	**23.2±0.51**	**0.20±0.01**
**High Yield**						
Dular	7.7±0.29	0.326±0.08	1267.16±91.8	62.7±5.5	24.9±0.59	0.18±0.00
Gontra Bidhan	8.4±0.32	0.306±0.01	2226.52±150.3	56.1±7.0	23.0±0.11	0.16±0.00
Way Rarem	8.3±0.26	0.538±0.03	1732.53±41.2	78.3±7.7	26.7±0.29	0.22±0.00
Patchai Perumal	8.6±0.27	0.474±0.02	2572.56±126.6	122.0±6.1	28.2±0.26	0.24±0.00
Sahbhagi Dhan	8.8±0.14	0.496±0.05	2071.94±76.7	81.3±8.1	24.5±0.51	0.25±0.00
Indra Barani Dhan	9.1±0.27	0.355±0.01	2444.64±95.6	84.0±6.3	24.3±0.28	0.24±0.00
MTU1010	8.2±0.13	0.314±0.02	2169.15±128.8	56.8±8.0	30.3±0.11	0.23±0.00
Maudamani	8.6±0.36	0.482±0.01	3683.56±90.2	NA	26.1±0.40	0.20±0.00
**Range**	7.7-9.1	0.306-0.538	1267.16-3683.56	56.1-122.0	23.0 -30.3	0.16 -0.25
**Mean**	**8.5±0.25**	**0.41±0.03**	**2271.9±100.15**	**77.3±7.0**	**26.0±0.32**	**0.22±0.00**
**C.D.**	0.022	0.092	264.84	-	1.43	0.05
**C.V.**	1.71	19.44	8.4	-	6.58	10.83

Table S5.

Allele mining of sixteen (8-LY and 8-HY) contrasting rice genotypes. Tables shows list of genes in which SNPs were identified.

Alternate codons	Locus ID	Ghanteswari	Parijat	Anjali	Khao Daw Tai	RKVY-104	BAM 5850	BAM 4510	Ghati Kamma N.	Dular	Gotrabi Dhan	Way Rarem	Patchaiperumal	Indrabaruni Dhan	MTU 1010	Sahbhagi Dhan	Maudamani
AAA➔AAC	Os08g09230	AA	CC	AA	AA	CC	CC	CC	AA	CC	AA	CC	AA	CC	AA	CC	AA
GCC➔ACC	Os08g09230	GG	AA	GG	GG	GG	GG	AA	GG	AA	GG	AA	GG	AA	GG	AA	GG
TTT➔TCT	Os08g09230	TT	CC	TT	TT	TT	TT	CC	TT	CC	TT	CC	TT	CC	TT	CC	TT
GAT➔GGT	Os08g09230	AA	GG	AA	AA	AA	AA	GG	AA	GG	AA	GG	AA	GG	AA	GG	AA
CGT➔CAT	Os08g09230	GG	AA	GG	GG	AA	AA	AA	GG	AA	GG	AA	GG	AA	GG	AA	GG
GTC➔GAC	Os01g52250	AA	AA	AA	AA	AA	AA	AA	AA	AA	AA	AA	AA	AA	AA	AA	AA
CAT➔CCT	Os01g52250	CC	CC	CC	CC	CC	CC	CC	CC	CC	CC	CC	CC	CC	CC	CC	CC
AGT➔GGT	Os06g06560	TT	TT	TT	TT	CC	CC	CC	CC	CC	CC	TT	CC	TT	TT	TT	CC
CCG➔GCG	Os02g36700	GG	GG	GG	CC	GG	GG	GG	GG	GG	GG	GG	GG	GG	GG	GG	CC
GGC➔GCC	Os02g36700	CC	CC	CC	CC	CC	CC	CC	CC	CC	CC	CC	CC	CC	CC	CC	CC

Table S6.

List of genes of photosynthetic, starch synthesis and sucrose transport and used for study of expression profile.
The primers sequences were either designed or obtained from Takashi et al., 2005

SL no	Enzyme	Locus ID	Gene name	Primer sequence
1	Ubiquitin 5 [UBI5]	LOC_Os01g22490	OsUbi5_F	GAAGCACAAGCACAAGAAGGTG
			OsUbi5_R	CTGGTTGTAGACGTAGGTGAG
2	*RUBISCO small subunit 2* [RBCS2]	LOC_Os12g17600	RbcS2_F	GAGGAGTCTGGTGGCAACTAAG
			RbcS2_R	CATCAAGTCAGAGTGAGATCAGGTG
3	*RUBISCO ACTIVASE 2* [RCA2]	LOC_Os11g47970	RCA2_F	ATCGTCAAGATCGTCGACAGCTTC
			RCA2_R	CTAACCGTAGAAGGAACCAGTCTTC
4	*FRUCTOSE-1,6-BISPHOSPHATASE* 1 [FBPase1]	LOC_Os06g40640	FBPase1_F	CTTTCACAGGCAAAGAACGGGTAC
			FBPase1_R	CATGCATGATTCGTCAGGCACTTC
5	*ZEAXANTHIN EPOXIDASE* 1 [ZEP1]	LOC_Os04g37619	ZEP1_F	TACCGACAACGAAGGTAGACG
			ZEP1_R	GACTGAAGTCTCTCGTTCATGCTG
6	Soluble starch synthase 4A [SSS4A]	LOC_Os01g52250	SSS4A_F	GGATAGACCAGACAGGATGCAAGAC
			SSS4A_R	TCCATGTTCCTGTGTCGATTCCAT
7	Soluble starch synthase 2B [SSS2B]	LOC_Os02g51070	SSS2B_F	CTCAACACGTACCGGAACTACAAGG
			SSS2B_R	TTCACTTCCACCGTTGCTCCTAC
8	Soluble starch synthase 4B [SSS4B]	LOC_Os05g45720	SSS4B_F	GGCTCAAGTCCAGTACCACACA
			SSS4B_R	CACAGAACCATATCTCATGGCAATC
9	Granule bound starch synthase 1 [GBSS1]	LOC_Os06g04200	GBSS1_F	ACGTCAGCGAGTGGGATCCTA
			GBSS1_R	TGCCGATGAACGCGATCAGT
10	Soluble starch synthase 1 [SSS1]	LOC_Os06g06560	SSS1_F	CATGGGAGGTCACAACTGCTGA
			SSS1_R	CAGAGGCACATCGGGCCTTATAG
11	Granule bound starch synthase 2 [GBSS2]	LOC_Os07g22930	GBSS2_F	CGGTCCTACCACTGGAGATGACTA
			GBSS2_R	GGACTCCAGTGTGCCAGTCAT
12	Soluble starch synthase 2C [SSS2C]	LOC_Os10g30156	SSS2C_F	GGAACTTGGTCTCCCAGTTCGT
			SSS2C_R	TATCTTCGAGGTCTTGCCGTCCA
13	Soluble starch synthase 3A [SSS3A]	LOC_Os08g09230	SSS3A_F	GCACCAGTTGCCTGGCTATACAAG
			SSS3A_R	ATGAGGAGCTATGGCACCATGAC
14	Soluble starch synthase 2A [SSS2A]	LOC_Os06g12450 Repeat below	SSS2A_F	AGCACTACCTGGATCACTTCAAGC
			SSS2A_R	GTTCTCCCGTATGATGTCGTGGAG
15	ADP-GLUCOSE PYROPHOSPHORYLASE SMALL SUBUNIT 2 [AGPS2]	LOC_Os08g25734	AGPS2_F	GCACGAGTGTGCTTGGAATCATTC
			AGPS2_R	CTCTTGACAGGTGACGGTTCAGAG

16	ADP-GLUCOSE PYROPHOSPHORYLASE SMALL SUBUNIT 1 [AGPS1]	LOC_Os09g12660	AGPS1_F	GGTTGGACTCCGGTCCTGTA
			AGPS1_R	CCAATGGGAATGCCACCAGTCT
17	ADP-GLUCOSE PYROPHOSPHORYLASE LARGE SUBUNIT 2 [AGPL2]	LOC_Os01g44220	AGPL2_F	CGAGCTTCTGACTATGGACTGGT
			AGPL2_R	TTGCATAGCTGAGGAAGCTGGTA
18	ADP-GLUCOSE PYROPHOSPHORYLASE LARGE SUBUNIT 1 [AGPL1]	LOC_Os03g52460	AGPL1_F	GGATAGGTTCCAATGTGCACCTC
			AGPL1_R	CACCTCAGATGACTAATCCATCTGC
19	ADP-GLUCOSE PYROPHOSPHORYLASE LARGE SUBUNIT 3 [AGPL3]	LOC_Os05g50380	AGPL3_F	ACCATGATGATGGGTGCGGATT
			AGPL3_R	TGTGATGACCACGTTCCTTCCA
20	ADP-GLUCOSE PYROPHOSPHORYLASE LARGE SUBUNIT 4 [AGPL4]	LOC_Os07g13980	AGPL4_F	GATGCTGTCAGGCAGTTCCTG
			AGPL4_R	CAGAAGCTCGACTCTCATCCACA
21	Sucrose phosphate synthase 1 [SPS1]	LOC_Os01g69030	SPS1_F	GAGATCATGCGTGCTCTCAAGG
			SPS1_R	CCAACAGTAGCATCAGCAGTG
22	Sucrose phosphate synthase 2 [SPS2]	LOC_Os02g09170	SPS2_F	GGCATTGAGGGTTACAGTACAGATG
			SPS2_R	GCTCAGGAATGGAACACACACAC
23	Sucrose phosphate synthase 3 [SPS3]	LOC_Os06g43630	SPS3_F	GTGGTGGTTCTTGTTGGTGAAAC
			SPS3_R	TGCTGTCTTTGTATCGTCCACTG
24	Sucrose phosphate synthase 4 [SPS4]	LOC_Os08g20660	SPS4_F	GACAGTGGTTGTTGGTGAAAGTGG
			SPS4_R	AGTAGTGTGTGTGCATACCTACTTG
25	Sucrose transporter 1	LOC_Os03g07480	SUT1_F	CGTCCTCAACATCTCCATCGTCAT
			SUT1_R	GGAGATCTTGGGCAGCAGGA
26	Sucrose transporter 2	LOC_Os12g44380	SUT2_F	ACTGCCCTCACATGGATTGGATG
			SUT2_R	CTTGGATCATCTGGACTTCCACGAT
27	Sucrose transporter 3	LOC_Os10g26470	SUT3_F	GCTGCTCGACTTCTCCAACAACA
			SUT3_R	CCAAGGATGTTGCCTAATGCCAT
28	Sucrose transporter 4	LOC_Os02g58080	SUT4_F	AGCAATGTCCCAGCTAACTCCAA
			SUT4_R	CAGGAGGTAGATGCCTCATGCTAG
